# Evaluation and clinical significance of serum neurospecific enolase in children with pneumonia: a case-control study

**DOI:** 10.1186/s12887-024-04852-6

**Published:** 2024-05-31

**Authors:** Tianhua Li, Minglei Li, Jie Feng, Tingting Liu, Liu Yang, Lexiang Yu

**Affiliations:** 1 Department of Paediatrics, Weifang People’s Hospital affiliated to Shandong Second Medical University, 151 Guangwen Road, Weifang, 261041 Shandong China; 2 Department of Ultrasound, Weifang People’s Hospital affiliated to Shandong Second Medical University, 151 Guangwen Road, Weifang, 261041 Shandong China

**Keywords:** Neurospecific enolase, Pneumonia, Lung disease, Children, Clinical significance

## Abstract

**Background:**

Neurospecific Enolase (NSE), a multifunctional protein, is present in various tissues of the body and plays an important role in many disease processes, such as infection, inflammation, tumours, injury, and immunity. In recent years, the application of NSE in respiratory diseases has become increasingly widespread and a research hotspot.

**Objective:**

This study aims to explore the relationship between NSE and childhood pneumonia, providing assistance for the diagnosis and assessment of pneumonia.

**Methods:**

Using prospective research and case-control methods, We selected 129 children with pneumonia hospitalised in Weifang People’s Hospital from September 2020 to April 2022 as the case group. Among them were 67 cases of Mycoplasma pneumoniae pneumonia (MP+), 62 cases of non-Mycoplasma pneumoniae pneumonia (MP -), and 21 cases of severe pneumonia. At the same time, 136 children who underwent outpatient health examinations were selected as the control group. The levels of NSE, ESR, CRP in cases group and NSE in control group were measured separately.

**Result:**

The NSE levels in the MP + group were 17.86 (14.29–22.54) ng/mL, while those in the MP- group were 17.89 (14.10–21.66) ng/mL, both of which were higher than the control group’s NSE levels of 13.26(12.18,14.44) ng/mL (H = 46.92, *P* = 0.000). There was no statistically significant difference in NSE levels between the MP + and MP - groups (*P* > 0.05). The NSE level in the severe pneumonia group was 27.38 (13.95–34.06) ng/mL, higher than that in the mild pneumonia group, which was 17.68 (14.27–21.04) ng/mL, (*P* = 0.024). The AUC values for diagnosing pneumonia are NSE0.714, CRP0.539, and ESR0.535, with NSE having the highest diagnostic value.

**Conclusion:**

Serum NSE can serve as an inflammatory indicator for paediatric pneumonia, which has important clinical guidance significance for the diagnosis, condition evaluation, and prognosis of paediatric pneumonia.

## Background

NSE is a cytoplasmic dimerase, an acidic protease unique to neurons and neuroendocrine cells and was first described by Moore and McGregor in 1965 [1]. It is mainly highly expressed in central nervous system tissue cells, followed by peripheral nerves and neuroendocrine cells [2, 3]. It has high application value in diagnosing neurological diseases in children. Under normal circumstances, the concentration of this enzyme in cerebrospinal fluid and blood is extremely low. When neuronal oedema, degeneration, and necrosis occur, NSE is released into the blood and cerebrospinal fluid through the damaged cell membrane and blood-brain barrier. The level of variation is positively correlated with the degree of brain injury. In the early stage, it is mainly used to evaluate the degree of brain injury and assist with the differential diagnosis of tumours, condition monitoring, efficacy evaluation, and recurrence prediction.

In recent years, dozens of neuroactive substances, such as Calcitonin gene-related peptide, 5-hydroxytryptamine, substance P, and Methionine enkephalin, which widely exist in central and peripheral nerve tissues, have been found [4, 5]. At the same time, various peptides or amines with biological activity have been found in the respiratory tract, and it is believed that they mainly come from non-adrenergic and non-cholinergic nerve fibres that innervate the airway — what some researchers refer to as the third nervous system [6]. Therefore, whether the lungs themselves can synthesise and secrete certain substances and whether there is a correlation between these bioactive substances and how these substances participate in the physiological and pathological processes of the lungs have attracted increasing attention from scholars. Further research suggests that [7], like the gastrointestinal tract, the lung is also an endocrine organ, and there is a group of endocrine cells called the lung neuroendocrine cells (PNECs). These cells have a special morphology, function, and close relationship with the nervous system. PNECs have unique neural and endocrine characteristics and can secrete a large number of enzymes and active substances, such as neurospecific enolase (NSE). PNECs are a very rich and diverse signal hub which produces a large number of neuropeptide and peptide hormones. They can directly send signals to many cells in the lung, to the brain through the lung sensory neuron, and possibly send signals to cells throughout the body through circulation [8].

The latest research suggests that [9] NSE has multiple functions, exists in various tissues, and cells, and participates in various reactions. In addition to its well-established glycolysis function in the cytoplasm, changes in cell localisation and differential expression of NSE are associated with several pathologies, such as infection, inflammation, autoimmune diseases, and cancer.

Pneumonia is one of the most important and common diseases in childhood, currently the leading cause of hospitalisation, especially Mycoplasma pneumoniae pneumonia, which poses a serious threat to children’s health [10]. There are many laboratory indicators for evaluating the severity of pneumonia, such as WBC, ESR, CRP, interleukin (IL), etc., but there is no specificity. NSE has been gradually applied to adult lung infectious diseases and is an important indicator used to evaluate the severity of pneumonia. However, there have been no reports on the utility of NSE in children with lung diseases. This study attempts to reveal the relationship between NSE and children’s infectious lung diseases by measuring the changes in serum NSE levels in children with pneumonia to aid in the clinical diagnosis and severity evaluation of pneumonia.

## Materials and methods

### Research object

Using a case-control study method, paediatric pneumonia patients hospitalised at Weifang People’s Hospital in Weifang City, Shandong Province, China, from September 2020 to April 2022 were selected as the case group. All included children needed to meet the diagnostic criteria for pediatric pneumonia in China [11] : Namely, cough, fever, shortness of breath, moderate to fine, moist rales on auscultation, and/or changes in keeping with pneumonia on chest X-ray or lung CT examination (opacities or small patchy shadows of varying sizes appearing in the lower and middle zones, or larger shadows, affecting segments). The enrolled children must be over 1 year old and able to complete all tests and examinations throughout the entire process. Children were excluded if they had an accompanying headache, vomiting, and/or abnormal mental state and suspected encephalitis. These children should have had electroencephalography, cranial magnetic resonance imaging (MRI), and/or other examinations. If necessary, a lumbar puncture was performed. If encephalitis was confirmed, the child was excluded from the case group. In addition, patients who had not completed their treatment and had been discharged or transferred early were also excluded from the case group. The case group of children was tested for Mycoplasma pneumoniae (MP) after admission and was divided into the MP + and MP- groups based on the test results. The diagnostic criteria for Mycoplasma infection included [12]: (1) A single serum MP antibody titre ≥ 1:160 (PA method); during the course of the disease, the double serum MP antibody titres increased by four times or more. (2) MP-DNA or RNA positive. The diagnostic criteria for severe pneumonia are shown in Table 1 [13]. Children who came as outpatients to the hospital at the same time as the children in the case group were randomly selected to form the control group. This study was approved by the ethics committee of Weifang People’s Hospital (No: P2021134). All of the parents of the children who participated signed an informed consent form.

**Table 1:** Evaluation criteria for severity of community acquired pneumonia in children

Evaluation items	Mild	Severe
General situation	Good	Not good
Disturbance of consciousness	No	Yes
Hypoxemia	No	Cyanosis; Faster breathing, RR ≥ 70 beats/min (infants), RR ≥ 50 beats/min (> 1 year old); Assisted breathing (moaning, nasal fan, triple concave sign); Intermittent apnea; Oxygen saturation < 92%
Fever	Not meeting the severity standard	Ultra high fever, sustained high fever > 5d
Dehydration syndrome/refusal to eat	No	Yes
Chest X-ray or chest CT	Not meeting the severity standard	≥ 2/3 unilateral lung infiltration, multilobed lung infiltration, pleural effusion, pneumothorax, atelectasis, lung necrosis, and lung abscess
Extrapulmonary complications	No	Yes
Standard	All of the above situations exist	Any of the above situations occur

Note: RR. Respiratory rate; Inflammatory indicators can serve as a reference for assessing severity

### Inspection method

All children participating in the testing had venous blood drawn from the cubital fossa vein on an empty stomach in the early morning and sent to our laboratory for NSE, CRP, and ESR testing. The detection of NSE was carried out using the Elecsys NSE assay kit (Roche, Basel, Switzerland) and the Roche fully automated electrochemical luminescence immunoassay system (Cobas e 601). The above procedures were carried out by professionals from the hospital laboratory.

### Statistics analysis

This study used SPSS25.0 software (IBM, Armonk, NY, USA) to analyse and process the experimental data. All sample data were subjected to normality and homogeneity of variance tests. The counting data is expressed in terms of examples (percentage), and inter-group comparisons were made using χ2 Inspection. If the measurement data conform to the normal distribution, it is expressed as mean ± standard deviation. The difference between groups was compared by t-test. The data with a non-normal distribution is expressed as median and Quartile (P25-P75). The comparison between the two groups was assessed with the Wilcoxon test. *P* < 0.05 indicates statistical significance. The correlation test between the two factors of skewed distribution was conducted using Spearman rank correlation analysis. *P* < 0.05 indicates statistical significance.

## Results

### Basic characteristics of the study subjects

A total of 129 children with pneumonia were included in the case group, including 78 males and 51 females, with an average weight of 22.0 ± 6.8 kg and an average age of 5.6 ± 1.7 years. There was no difference in gender and age between cases and controls (*P* = 0.15 and *P* = 0.13, respectively). There were 67 cases of MP+, 62 of MP-, 21 with severe pneumonia, and 108 with mild pneumonia in the case group. The clinical characteristics and other blood tests are shown in Table 2.

**Table 2:** Clinical characteristics of children with pneumonia(*N* = 129)

Characteristics	Value or percentage	Laboratory examination	Value or percentage
Boys (%)	78/129	WBC↑or↓	64/129
Age (year($\bar x \pm s$))	5.6 ± 1.7	CRP↑	53/129
Weight (kg($\bar x \pm s$))	22.0 ± 6.8	ESR↑	96/129
Course of Disease(day($\bar x \pm s$))	8.23 ± 4.12	ALT or AST↑	25/129
Severe pneumonia(%)	21/129	CK-MB↑	24/129
MP+(%)	67/129	PLT↑	16/129
MP-(%)	62/129	D-dimer ↑	15/129

ALT: Alanine aminotransferase, CK-MB: creatine kinase isoenzymes MB, CRP: C-reactive protein, ESR: erythrocyte sedimentation rate, MP+: Mycoplasma pneumoniae pneumonia, MP-: non Mycoplasma pneumoniae pneumonia, PLT: platelet, WBC: white blood cell,

### Serum NSE levels in cases and controls

The NSE levels in the pneumonia MP + group, pneumonia MP - group, and control group were 17.86 (14.29–22.54) ng/mL, 17.89 (14.10-21.66) ng/mL and 13.26(12.18,14.44) ng/mL, respectively. There were statistical differences in the overall distribution of NSE among the three groups (H = 46.92, *P* = 0.000). There was a statistically significant difference in NSE levels between the MP + group and the control group (*P* = 0.000) and between the MP- group and the control group (*P* = 0.000). There was no statistically significant difference in NSE levels between the MP + group and the MP- group (*P* > 0.05) (Table 3).

**Table 3:** Serum NSE levels in the experimental and control groups

Group	Median (*P*25, *P*75)	Rank-sum test	
		H value	*P* value
MP(+ )group	17.86(14.29,22.54)※	46.92	0.000
MP(- )group	17.89(14.10,21.66)※		
Controls	13.26(12.18,14.44)		

※compared with the control group, *P* < 0.05

### Serum NSE levels in the severe and mild pneumonia groups

The serum NSE level in the severe pneumonia group was 27.38 (13.95–34.06) ng/mL, 17.68 (14.27–21.04) ng/mL in the mild pneumonia group, and 13.26(12.18,14.44) ng/mL in the control group. There was a statistical difference in the overall distribution of NSE among the three groups (H = 51.94, *P* = 0.000). There was a statistically significant difference in NSE levels between the severe pneumonia group and the control group (*P* = 0.000), and between the mild pneumonia group and the control group (*P* = 0.000). There was also a statistically significant difference in NSE levels between the severe pneumonia group and the mild pneumonia group (*P* = 0.024) (Table 4).

**Table 4:** Serum NSE levels in the severe pneumonia group and the mild pneumonia group

Group	Median (*P*25, *P*75)	Rank-sum test	
		H value	*P* value
Severe pneumonia group	27.38(13.95,34.06)※	51.94	0.000
Mild pneumonia group	17.68(14.27,21.04)※		
Controls	13.26(12.18,14.44)		

※compared with the control group, *P* < 0.05

### The predictive ROC curve of serum NSE, ESR, and CRP for pneumonia

**Fig. 1:**
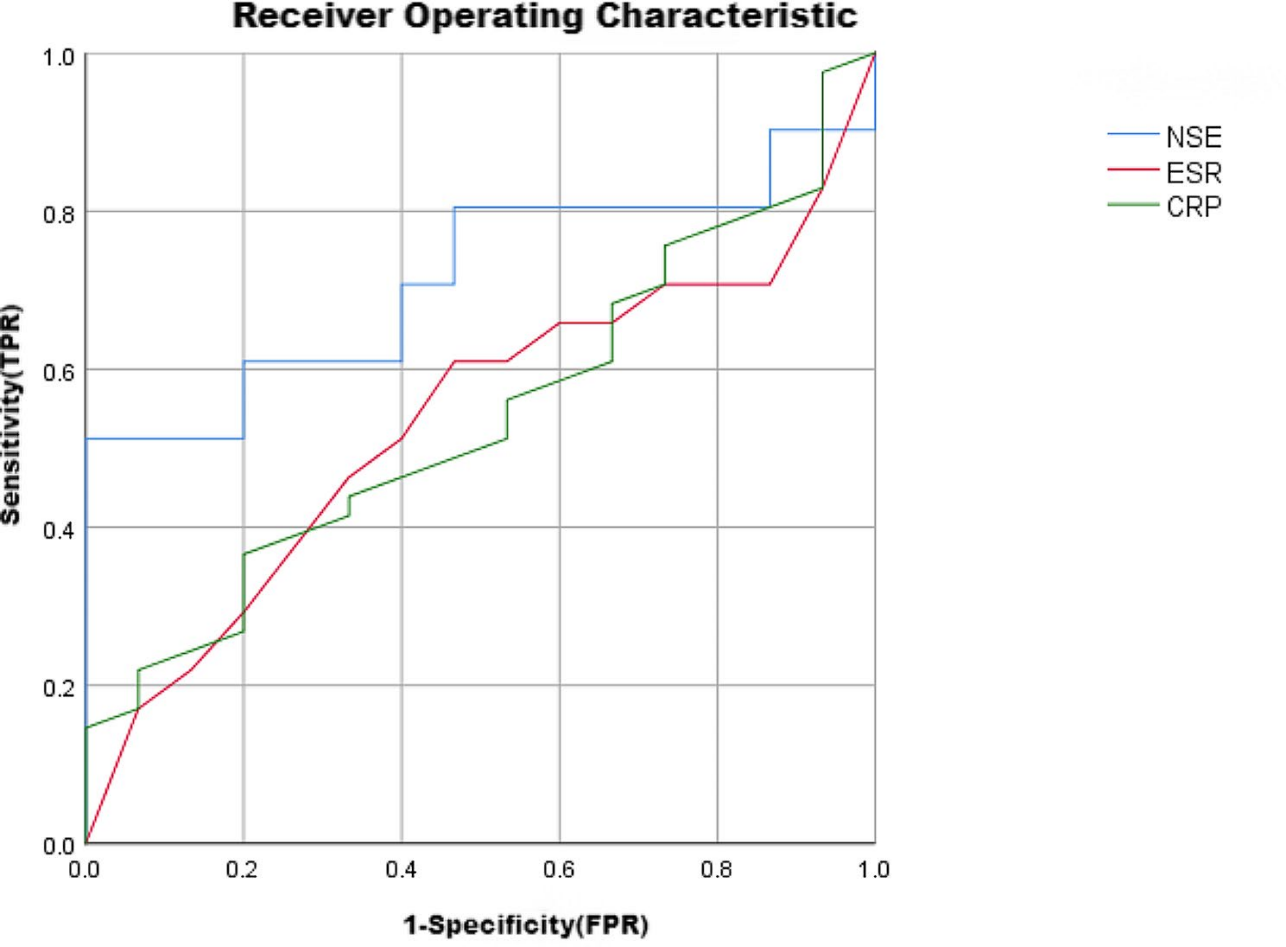
The predictive ROC curve of serum NSE, ESR, and CRP for pneumonia

Draw ROC curves for predicting pneumonia in children with NSE, ESR, and CRP in the control group. The AUC value for NSE diagnosis of pneumonia is 0.714; The AUC value for CRP diagnosis of pneumonia is 0.539; The AUC value for ESR diagnosis of pneumonia is 0.535. The diagnostic value of NSE is the highest, as shown in Fig. 1.

## Discussion

Pneumonia is the most common lung disease in children, with viral infections being the most common cause in young infants and bacterial infections being the most common cause in older children. In recent years, the incidence rate of MP infection in children has increased, and it is an important pathogenic microorganism that causes children’s community-acquired pneumonia (CAP) [14]. In addition, there is a large-scale epidemic in China every few years, which seriously affects children’s health. We conducted a study on 129 paediatric pneumonia patients admitted to Weifang People’s Hospital from September 2022 to April 2022 and found that there were 67 cases of MP, accounting for 52%, which has become the most common cause of pneumonia in hospitalised children in our hospital. MP produces the community-acquired respiratory distress syndrome toxin, likely leading to inflammation and airway dysfunction. The formation of virulence factors, such as lactoferrin, hydroxyl radicals, superoxide anions, and hydrogen peroxide, aggravate tissue damage. These factors can also cause other systemic symptoms, such as elevated serum IgE [15] and changes in coagulation [16].

We found that the serum NSE levels of children in the case group were higher than those in the control group. Moreover, the NSE levels in the severe pneumonia group were significantly higher than those in the mild pneumonia group. It suggests that there may be a specific correlation between NSE and the occurrence of pneumonia, which is directly proportional to the severity of pneumonia. This is consistent with the research findings in adult pneumonia. Inflammation, hypoxia, and other factors can cause the NSE serum levels to increase significantly. It can be used as an inflammatory indicator for paediatric pneumonia. However, there was no significant difference in NSE levels between the MP + and MP- groups and no significant correlation with erythrocyte sedimentation rate (ESR) and C-reactive protein (CRP). So, whether there are differences in NSE levels between different pathogen infections still requires further in-depth research.

The earliest application of NSE in lung diseases was in lung cancer, and it has been widely used and studied in depth, especially in small-cell lung cancer with neuroendocrine functions, where serum NSE levels are significantly elevated [17]. NSE has been used as a tumour marker for the diagnosis, prognosis, and follow-up of small cell lung cancer, and its serum levels vary significantly depending on tumour size, disease stage, and metastasis. However, Patricia et al. [18]. found a general increase in NSE in 178 patients with breast, lung, stomach, and kidney tumours; therefore, it is believed that NSE is not specific and is elevated in almost all patients with tumours and is considered a neuroendocrine marker. In recent years, research on NSE in lung diseases has gradually increased. According to the literature, NSE is involved in many lung diseases besides lung cancer, plays an important role in lung diseases, and has diagnostic potential. It is considered a bridge between the nervous system and the respiratory system [9].

NSE has important value in the diagnosis of pulmonary tuberculosis (TB). Racil et al. [19]. conducted a prospective study in 2009. They collected blood samples from 40 male patients with tuberculosis before anti-tuberculosis chemotherapy was started and measured the serum levels of four tumour markers, including NSE, cancer antigen 125 (CA125), angiotensin-converting enzyme (ACE) and CYFRA21-1. They found that 91.66% of cases had higher levels of NSE, significantly higher than other tumour markers. In addition, several studies have found that the levels of NSE, S100B, and Neuropeptide Y (NPY) in the serum and cerebrospinal fluid of children with acute miliary TB secondary to TB meningitis are significantly higher than those of children with acute miliary TB or meningitis alone [20, 21]. NSE is associated with chronic obstructive pulmonary disease (COPD). Barouchos et al. [22]. studied the correlation between tumour markers and inflammatory biomarkers in patients with COPD exacerbation. As a result, there was a significant positive correlation between CRP and CA125 and NSE and a significant positive correlation between ESR and NSE. Jie Li et al. [23]. studied 102 patients diagnosed with COPD in Nanchang Provincial Chest Hospital in 2017 and found that the serum NSE level of patients with COPD gradually increased with the increase in COPD severity.

NSE is associated with interstitial lung disease. Pulmonary alveolar proteinosis (PAP) is a rare interstitial lung disease characterised by the abnormal alveolar accumulation of surfactant components. The aetiology is unknown and may be related to alveolar macrophage dysfunction. Due to the lack of typical clinical symptoms, the diagnosis of PAP is difficult. Ludtke et al. [24]. studied the clinical, pathological, and biochemical characteristics of 11 patients with PAP. They observed that CEA was elevated in most patients, while CYFRA21-1 and NSE were elevated in all patients. Fang et al. [25]. also found a general increase in NSE in their study of PAP patients, consistent with Ludtke’s study. Arai et al. [26]. found that the changes in tumour markers such as CEA, SCC, and NSE in the serum of PAP patients were consistent with the changes in disease severity indices such as lactate dehydrogenase (LDH), PaO2 and so on. In addition, Zhao et al. [27]. conducted a study on 455 cases of silicosis diagnosed at Yantai Mountain Hospital from January 2018 to December 2019 and found that serum NSE levels were significantly elevated in silicosis patients, which can serve as an important reference indicator for the diagnosis and differential diagnosis of silicosis.

There are few studies on the correlation between NSE and pneumonia in the literature, with only a few reports on adults. Erika Cione et al. [28]. evaluated the serum NSE level of patients with and without dyspnoea with coronavirus disease 2019 (COVID-19) in a study on adult COVID-19. These were adult patients (> 18) with severe acute respiratory syndrome coronavirus 2 infection who were referred to Catanzaro, Italy, from March 30 to July 30, 2020. The NSE value of patients with COVID-19 was significantly higher than that of the control group in this study.

Denise et al. [29] found, in their retrospective study of COVID-19, that NSE and other biomarkers such as LDH, aspartate aminotransferase (AST), troponin, creatine kinase (CK-MB), D-dimer, and brain natriuretic peptide (BNP) can easily be used to predict the severity of disease, hospitalisation, admission to the intensive care unit (ICU), and mortality. These data provide strong evidence for further research on NSE as a clinical marker of pneumonia progression.

How does NSE play a role in lung infection or injury? As is well known, various serious diseases can cause acute lung injury (ALI), such as sepsis, severe acute pancreatitis (SAP), acute respiratory distress syndrome (ARDS), ketoacidosis, etc. We believe that NSE, as an essential enzyme in the glycolytic pathway (a key isoenzyme of glycolysis), can promote the production of active substrates such as reactive oxygen species, NO, and various inflammatory mediators such as TNF-α, IL-1 β, INF- γ, TGF- β, and MCP-1 through pro-inflammatory signalling pathways, thus playing a crucial pathogenic role in ALI [30, 31]. Lawrence’s research team [32] found that in a rat ALI model induced by SAP, the expression of NSE, caspase-1, IL-1β, and TNF-α in injured rat lung tissue were significantly increased. Applying NSE inhibitors to inhibit the glycolytic pathway can significantly inhibit the expression and activation of caspase-1, reducing lung injury. The findings of Capello et al. [33]. also confirmed that NSE is significantly elevated in acute and chronic lung injury. Gong et al. [34]. conducted an experiment in which they attacked mouse pulmonary endothelial cells with lipopolysaccharide (LPS) and found a significant increase in the expression and activity of a key glycolytic activator, PFKFB3. This may be related to the abnormal enhancement of glycolysis mediated by NSE. Zhong et al. [35]. studied mice with acute ALI induced by LPS and found that inhibiting the glycolytic pathway can alleviate the onset of acute ALI induced by LPS.

## Conclusions

In conclusion, as a multifunctional protein, NSE plays an important role in many lung diseases, including paediatric pneumonia. It plays an important role in the diagnosis, disease monitoring, and prognosis evaluation of paediatric pneumonia and can be used as an inflammatory indicator for children’s infectious lung diseases. Due to the lack of previous reports on the correlation between NSE and pulmonary diseases in children, this study is a first. The sample may not be representative as it was conducted in a single institution. Additionally, the sample size may not be large enough, and some unknown factors may have influenced the results. We hope that more paediatricians and more hospitals will join this study.

## Data Availability

The data that support the findings of this study are available from the author Lexiang Yu upon reasonable request.
